# Influence of HIV Infection and Antiretroviral Therapy on Bone Homeostasis

**DOI:** 10.3389/fendo.2020.00502

**Published:** 2020-09-02

**Authors:** María Victoria Delpino, Jorge Quarleri

**Affiliations:** ^1^Instituto de Inmunología, Genética y Metabolismo (INIGEM), Universidad de Buenos Aires, CONICET, Buenos Aires, Argentina; ^2^Instituto de Investigaciones Biomédicas en Retrovirus y Sida (INBIRS), Universidad de Buenos Aires, CONICET, Buenos Aires, Argentina

**Keywords:** HIV, HAART, bone, osteoblast, osteoclast

## Abstract

The human immunodeficiency virus type 1 (HIV)/AIDS pandemic represents the most significant global health challenge in modern history. This infection leads toward an inflammatory state associated with chronic immune dysregulation activation that tilts the immune-skeletal interface and its deep integration between cell types and cytokines with a strong influence on skeletal renewal and exacerbated bone loss. Hence, reduced bone mineral density is a complication among HIV–infected individuals that may progress to osteoporosis, thus increasing their prevalence of fractures. Highly active antiretroviral therapy (HAART) can effectively control HIV replication but the regimens, that include tenofovir disoproxil fumarate (TDF), may accelerate bone mass density loss. Molecular mechanisms of HIV-associated bone disease include the OPG/RANKL/RANK system dysregulation. Thereby, osteoclastogenesis and osteolytic activity are promoted after the osteoclast precursor infection, accompanied by a deleterious effect on osteoblast and its precursor cells, with exacerbated senescence of mesenchymal stem cells (MSCs). This review summarizes recent basic research data on HIV pathogenesis and its relation to bone quality. It also sheds light on HAART-related detrimental effects on bone metabolism, providing a better understanding of the molecular mechanisms involved in bone dysfunction and damage as well as how the HIV-associated imbalance on the gut microbiome may contribute to bone disease.

## Introduction

According to UNAIDS, 37.9 million people worldwide are currently living with HIV/AIDS and about 22 million are on highly active antiretroviral therapy (HAART). The life expectancy of HIV-infected individuals treated with HAART is nearly normal, with a decreased incidence in AIDS-related morbidity and mortality (1).

Low bone mineral density (BMD) has frequently been observed among HIV-infected individuals, likely leading to osteopenia and osteoporosis with a high prevalence of fractures compared with the general population (2).

In HIV-infected patients, bone loss is primarily enhanced by two pivotal factors: HIV infection and its direct consequences, and HAART, mainly during the first years of treatment (3–8). The contribution of each one is still controversial. The evidence of reduced bone mass in treatment-naïve patients indicates that the virus alone directly affects bone homeostasis (9–14). Moreover, some reports indicate that low BMD is not completely attributable to HIV infection alone or HIV infection plus treatments with HAART (15–23).

The bone as part of the skeletal system interacts with immune cells in the bone marrow, interacting with each other in a significant mutual influence (24). Recently, the molecular mechanisms involved in the homeostatic interactions between bone and immune cells has been elucidated (25–27), which HIV appears to be able to disturb.

## Microbiota Contribution in HIV Interaction With Bone

### HIV Proteins

HIV genes encode regulatory, auxiliary, and structural proteins. The regulatory proteins include the HIV trans-activator (Tat) involved in the regulation of the reverse transcription of the viral genome, and the regulator of expression of virion proteins (Rev) responsible for the synthesis of major viral proteins. The auxiliary HIV proteins comprise the negative factor (Nef), which is implicated in multiple functions during the viral replication cycle, including, among other functions, the lentivirus protein R (Vpr) responsible for nuclear import of the pre-integration complex. It is also comprised of the viral infectivity factor (Vif) required to synthesize infectious viruses in several human cells and the virus protein U (Vpu) the main role of which is the successful release of virions from infected cells. The structural proteins included the group-specific antigen (p55 gag polyprotein), a polyprotein which is processed by viral proteases during maturation to matrix protein (p17), capsid protein (p24), spacer peptide 1 (p2), nucleocapsid protein (p7), spacer peptide 2 (p1), and P6 protein. Other structural proteins involve the polymerase (Pol) and the envelope protein (gp160) that is post-translationally processed to produce the surface glycoprotein (gp120) and gp41 that mediate binding to the CD4 receptor, and envelope fusion to target cells, respectively (28).

### Interaction of HIV and Its Proteins With Bone Cells

Among many of the viral pathogenic mechanisms, HIV regulatory, auxiliary, and structural proteins play critical roles during cell-host interaction and thus have shown significant impacts on bone in experimental studies, promoting changes in the balance of bone formation and resorption. It is important to highlight that the HIV-induced detrimental effects on cells are not only a consequence of the active viral replication and the role of infectious virions but are also caused by several HIV proteins that are released to extracellular media which could induce bystander harmful effects, such as apoptosis, oxidative stress, mitochondrial dysfunctions, or autophagy alterations, on surrounding cells (29, 30).

Mesenchymal stem cells (MSCs) are multipotent precursors able to differentiate toward multiple tissue lineages such as adipocytes, chondroblasts, and osteoblasts (31, 32). As MSCs express CD4 receptors and CCR5 and CXCR4 coreceptors, these cells are likely susceptible to HIV infection, although integrated proviruses are rarely found and productive infection has not yet been documented (33). Nonetheless, hematopoietic progenitor cells (HPCs) in the bone marrow of HIV-infected individuals have been regarded as a persistent HIV reservoir (34).

Differentiation of MSCs *ex vivo* into both osteoblasts and adipocytes depicted a dichotomy upon exposure to the serum source, since those in contact with a high HIV viral load preferentially acquired a proadipogenic phenotype whereas those in contact with low viral load serum were induced toward an osteogenic condition. This phenomenon may involve Tat protein, which inhibits the transcription factor COUP TF-I (chicken ovalbumin upstream promoter transcription factor), thus favoring adipocyte differentiation while preventing osteoblast development.

To command the balance of bone resorption and formation, osteoblasts produce a receptor activator factor of nuclear factor-kB ligand (RANKL) that controls the differentiation of osteoclasts (35). Osteocytes -the terminally differentiated form of osteoblast- also produce RANKL to regulate osteoclast activity (36). Under physiological conditions, osteoclastogenesis involves RANKL and macrophage colony-stimulating factor (M-CSF) produced by osteoblast and bone marrow stromal cells (37). M-CSF prompts the expression of RANKL receptor (RANK), on osteoclast precursor which then interacts with RANKL to initiate osteoclasts' differentiation (38). As a counterpart, osteoprotegerin (OPG) is a neutralizing soluble trap receptor expressed by bone marrow stromal cells and osteoblasts able to inhibit the RANKL-RANK interaction (39).

The Tat protein enhances peripheral blood monocyte-derived osteoclast differentiation and activity by RANKL plus M-CSF treatment, which increases both the mRNA transcription of specific osteoclast differentiation markers, such as cathepsin K and calcitonin receptor, and the tartrate-resistant acidic phosphatase (TRAP) expression and activity. Together, these results show that Tat may be considered a viral factor that stimulates osteoclastogenesis and bone resorption activity (11, 40–42). *In vitro*, Tat and Nef proteins reduce -in a cumulative manner- the number of bone marrow MSCs available to differentiate into osteoblasts by inducing early senescence, associated with increased oxidative stress and mitochondrial dysfunction of these cells. Moreover, Tat, but not Nef, induced an early increase in NF-κB activity and cytokine/chemokine secretion, and reciprocally, Nef- but no Tat-treated cells -have shown early autophagy inhibition (43).

The HIV accessory protein Vpr upregulates the RANKL expression in peripheral mononuclear cells from healthy donors, enhancing osteoclastic activity. This action is synergized by both exogenous and endogenous glucocorticoids as a potent cofactor in bone mineral loss (44). Moreover, Tat and Rev proteins increase monocyte differentiation into osteoclasts, as well as boost osteoclast resorption function by increasing reactive oxygen species and TNF-α production in osteoclast precursors (45, 46).

In an *in vivo* humanized mice and *ex vivo* human joint tissue study, Raynaud-Messina et al. have contributed to our current understanding of the HIV-induced bone loss mechanisms. For the first time, the authors demonstrated that HIV infects osteoclast precursors even at different stages of osteoclastogenesis, either via cell-free viruses or, more efficiently, through transfer from infected T cells. These infected precursor cells have been proposed as HIV reservoirs that display a greater migratory capacity and exhibit the enhanced ability to recruit and concentrate in the bones where the viral infection alters the bone resorption machinery. HIV can enlarge podosomes and enhance the osteolytic activity of the bone resorption apparatus, also known as the “sealing zone” (SZ). The virus is also able to increase the TRAP secretion by osteoclasts, leading to demineralization and degradation of larger bone extensions. These viral-directed actions are Nef-mediated and are abundantly produced and secreted during the early phase of viral replication. Such Nef-mediated actions occur through the activation of Src, which regulates podosomes into the SZ (47).

Soluble HIV-structural proteins are also mediators of cytopathogenic effects. These proteins may act as part of the viral particle or as bystander effect mediators after their release from productively infected cells (48). Both, p55-gag and gp120 were found to reduce calcium deposition, alkaline-phosphatase activity, levels of secreted BMP-2, -7, and RANKL, as well as the expression and activity of the pro-osteogenic transcription factor runt-related transcription factor 2 (RUNX2) in human osteoblasts. The levels of osteocalcin were also significantly reduced by p55-gag treatment, while gp120 also increased the pro-adipogenic transcription factor and peroxisome proliferator-activated receptor γ (PPARγ) activity. The ability of MSCs to develop into functioning osteoblasts was also affected by the presence of HIV proteins, with p55-gag inducing a decrease in osteogenesis, while rev induced an increase (49). A positive feedback loop exists between RANKL production and HIV replication, which may be relevant to both the pathophysiology of HIV-linked osteopenia and the control of HIV replication (50).

Furthermore, HIV gp120 can trigger *in vitro* osteoblast apoptosis induction mediated by the up-regulation of TNF–α (51). In these cells, gp120 enhances the expression of Dickkopf-1 (Dkk1), the antagonist of the Wnt, significantly reducing the intracytosolic and intranuclear β-catenin expression, the alkaline phosphatase activity, and the cell proliferation (52).

In HIV-infected individuals, B and T lymphocytes have exhibited several signs of dysfunction with an impact on bone homeostasis. They are sources of OPG and, consequently, their dysfunction contributes to viral-induced bone loss. Hence, there is a higher frequency of RANKL-expressing B cells (resting memory and exhausted tissue-like memory B cells) expanded as a consequence of inflammation and a lower frequency of OPG-expressing B cells (resting memory B cells) in HIV-infected compared to HIV-uninfected individuals, thus resulting in a lower RANKL/OPG ratio that correlates with total hip BMD, T-, and Z-scores in the HIV-infected participants (14).

**Figure 1:**
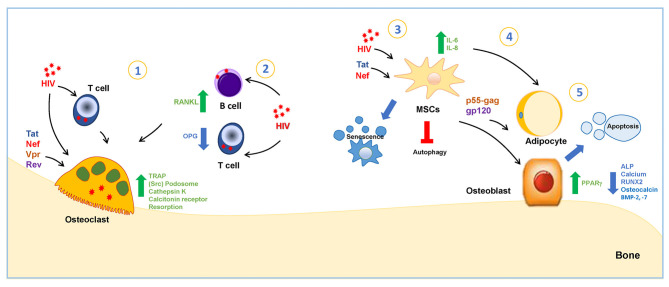
HIV and viral proteins' interaction with bone cells. **1**-HIV infects osteoclasts via cell-free viruses or by cell-to-cell transfer from infected T cells. The infection increases the tartrate-resistant acidic phosphatase (TRAP) secretion by osteoclasts. The viral protein Tat increases mRNA transcription of cathepsin K, calcitonin receptor, TRAP, and Nef-regulate podosomes through activation of Src. Vpr upregulates RANKL expression, stimulating osteoclastogenesis. Tat and Rev increase osteoclastogenesis. **2-**HIV infection induces an increase in RANKL-expression and the reduction of OPG-expression in B and T cells. **3-**HIV proteins Tat and Nef reduce the number of bone marrow MSCs by inducing early senescence. Tat stimulates MSC to secrete IL-6 and IL-8, and Nef induces the inhibition of autophagy. **4-**Human serum with a high HIV viral load preferentially acquired a proadipogenic phenotype in a mechanism dependent on Tat protein, while those in contact with a low viral load serum were induced toward osteogenic conditions. **5-**p55-gag and gp120 stimulate osteoblast apoptosis and reduce alkaline-phosphatase activity (ALP), calcium deposition, the runt-related transcription factor 2 (RUNX-2), and Bone morphogenic protein-2 and−7 (BMP-2−7), and p55-gag also reduces osteocalcin levels, and gp120 induces the increase in peroxisome proliferator-activated receptor γ (PPARγ).

Similarly, T-cell OPG production was also significantly lower in CD4 T-cell-sufficient HIV-infected individuals (>200 cells/μl) but not in those with lower cell counts. It was coupled with moderately higher T-cell RANKL production, resulting in a significantly higher T-cell RANKL/OPG ratio. Such a T-cell RANKL/OPG lowered ratio correlated significantly with BMD-derived z-scores at the hip, lumbar spine, and femur neck (53). Moreover, as a bystander effect, such an abnormal RANKL expression by T cells is mimicked when these cells are exposed to soluble gp120 (Figure 1) (54).

### HIV-Related Gut Microbiome Alterations and Its Relationship With Bone Loss

Recently, the gut microbiota has been reported to have an influence on bone metabolism, attracting attention as a prospective new target to balance BMD. The basis of this evidence is mainly concentrated on its involvement in modulating the interface between the immune system and bone cells (55, 56).

As an early event, the gut microbiome in HIV-infected individuals exhibits different compositions compared to uninfected individuals (57, 58). Among them, the bacterial composition is altered on its diversity, genes, and functional capabilities, that are either pro-inflammatory or potentially pathogenic and whose abundance correlated with immune status (59, 60). T-cell depletion is pronounced at the gut-associated lymphoid tissue (GALT) promptly after HIV infection, followed by an increase in the barrier permeability and microbial translocation with increased LPS levels (61). This context induces an innate immune activation leading to a shift toward a pro-inflammatory cytokine environment with osteoclastogenesis and bone resorption enhancement (62, 63).

Since chronic immune activation with progressive immune suppression impacts on the gut microbiome, a differential contribution of gut bacteria and their molecular agents (metabolites and proteins) is desirable to promote immune recovery in HIV-infected individuals. Hence, after characterizing the interplay between the active gut microbiota and the host, it is plausible to reduce inflammation and recover the immune–skeletal interface (64–66).

The HAART treatment effect on gut microbiota in HIV patients is uncertain (67). One hypothesis is that HIV treatment stimulates the restoration of normal microbial flora (68). However, some studies show a minimal effect of HAART on the restoration of normal microbial flora (68–70) while others reveal a negative impact (71).

## Role of Antiretroviral Therapy on Bone Tissue Metabolism

The widespread accessibility of HAART has changed HIV from a life-limiting condition to one with a near-normal life expectancy. Unexpectedly, throughout such a therapy, the bone loss promoted by HIV-infection may continue unabated. However, among HIV-infected individuals on HAART, the presence of osteoporosis appears to be about three times higher than those uninfected (3–8). Although, far from consensus, other reports have estimated up to a 6% decrease in BMD upon HAART treatment initiation for a 2-year period, but then the BMD remains unchanged despite continuing therapy (72–74).

As mentioned above, in naïve immunosuppressed HIV-infected individuals a decrease in BMD is observed. Paradoxically, when these individuals are on HAART they achieve their immune reconstitution by CD4+ T cell repopulation (75, 76). These reports offer evidence of stable or increasing BMD with plausible early, but small and not sustained, loss of BMD that accompanies the initiation of HAART, and without accelerated bone loss in the medium term (77–82).

The gender of the HIV-infected individual also influences the BMD reduction grade. Among HAART-treated patients, it appears to be more accentuated in women than in men (83, 84), but is at a level similar to that observed initially during menopause (85).

Several studies have directly emphasized HIV factors associated with low BMD: duration of infection, HIV viral burden, and a more advanced HIV disease (86–88). In this regard, data presented in a sub-study of the Strategy for Management of Antiretroviral Therapy (SMART) study demonstrated a low level of bone turnover markers but higher BMD when HAART is interrupted, thus inferring a higher HIV RNA level and lower CD4+ T cell counts (89). In contrast, Grund et al. have reported that continuous HAART was associated with significant reductions in BMD with no changes or increases in BMD observed in those on intermittent ART (90). Similarly, longitudinal data collected from randomized control trials have insinuated that the initiation of HAART at higher viral RNA and lower CD4+ T cell counts at baseline were associated with more pronounced reductions in BMD (88). Such low pre-treatment CD4 counts were reported as a strong and independent risk factor for loss of BMD during treatment. However, loss of bone continues for up to 2 years after HAART initiation and the extent of immune reconstitution was not related to BMD improvement (88). In conjunction, these data suggest that important roles are played directly by HIV and/or indirectly by the immune response in BMD loss.

The effect of HAART on BMD seems to be influenced by the specific type of treatment. Low BMD has been associated with regimens such as nucleoside analog reverse-transcriptase inhibitors (NRTIs) (74, 91, 92). Individuals exposed to tenofovir disoproxil fumarate (TDF)-based treatment in particular exhibited a more accentuated BMD loss compared to individuals on other regimens, such as lamivudine (3TC) and emtricitabine (FTC), or those who have been switched to two-drug regimens (74, 91–97). However, others have reported contradictory findings regarding TDF-therapy duration and BMD loss, even after long-term exposure to the drug (98).

The underlying mechanisms by which antiretroviral drugs promote BMD loss are still controversial. The mechanism to NRTIs-mediated BMD loss may be promoted by elevated lactic acid concentration in the blood leading to calcium hydroxyapatite loss, especially in the trabecular bone, due to the labile of calcium storage (99). Regarding the underlying mechanisms that may be related to TDF-associated lower BMD, mitochondrial toxicity, hyperphosphaturia secondary to tubular dysfunction, and renal osteodystrophy have been considered (92, 100–102). Despite the bone loss, there are contradictory findings about phosphate metabolism abnormalities observed among HAART-treated individuals who can present higher phosphate blood levels and lower bone density (86). These data offer supportive information to avoid the use of TDF and its replacement with bone-friendly regimens among the HIV-infected population with fracture risks (103).

Besides the BMD reduction related to NRTIs, available data regarding protease inhibitors (PIs) remain contradictory (104). On the one hand, increased bone turnover, accelerated bone loss, and a higher prevalence of reduced BMD have been reported (3, 72, 92, 105–107), whereas other studies showed opposed results (9, 10, 73, 108). Detrimental effects on BMD are in line with *in vitro* observations evaluating the effect of different PIs on osteoblast activity (109). For example, pharmacologic levels of two PIs that are clinically linked to osteopenia, ritonavir (RTV) and saquinavir (SQV) but not indinavir (IDV) and nelfinavir (NFV), abolish the interferon-γ-mediated degradation of the RANKL signaling adapter protein TRAF6 (tumor necrosis factor receptor-associated protein 6) in proteasomes. Moreover, under inflammatory conditions, interferon-γ promotes bone loss mainly by up-regulating the activity of macrophages, leading to T cell activation and osteoclastogenic cytokine production (110).

RTV appears as an osteoclast-activating agent that promotes the proliferation and activation of osteoclasts *in vitro* (111, 112) and *ex vivo* studies (113), causing increased bone absorption.

Importantly, most of these *in vitro* direct effects of PIs on bone cells did not resemble the *in vivo* observations collected from patients on HAART. RTV, SQV, and fosamprenavir (FPV) appear to improve the BMD *in vitro* rather than the loss observed *in vivo*, by decreasing RANKL and increasing OPG secretion (54, 109). The impact on BMD loss was also reported in several *in vivo* studies which also observed a strong difference in bone loss according to PI discontinued and continued schemes between patients (72, 92, 107). RTV -but not IDV- at a greater than normal concentration was able to inhibit osteoclast function and suppress osteoclastogenesis *in vitro* and *in vivo* by impairing RANKL-induced signaling (114). However, RTV at plasma concentration, as a PI-boosting drug, favors the differentiation of blood monocytes into osteoclasts by up-regulating the production of transcripts for osteoclast growth factors using the non-canonical Wnt proteins 5B and 7B as well as activated promoters of nuclear factor-kappaB signaling, but suppressing genes involved in canonical Wnt signaling. Additionally, RTV blocks the cytoplasmic-to-nuclear translocation of β-catenin, the molecular node of the Wnt signaling pathway, in association with enhanced β-catenin ubiquitination (111, 112). *In vivo*, among RTV-treated patients, its discontinuation resulted in a slower decrease in BMD (107), and the bone mineral loss appeared in a time-dependent manner irrespective of dosage (107). Other PIs, such as IDV and NFV, have been shown to have a negative impact on osteoblasts by impairing its alkaline phosphatase activity and calcium deposition. Lastly, *in vivo* and *in vitro* studies demonstrate that PIs atazanavir (ATV) and lopinavir (LPV) also decrease BMD by impairing the MSCs differentiation to osteoblasts (72, 92, 115).

Finally, in addition to immune cells, the HIV-coreceptor CCR5 has been involved in the regulation of the function of bone cells by directly modulating osteoclastogenesis and the communication between osteoclasts and osteoblasts (116–118). In this regard, epidemiological evidence suggests that the functional loss of CCR5 is correlated with a lower incidence of bone-destructive diseases as well as of HIV transmission. Using a CCR5-deficient murine model, the osteoclasts appeared dysfunctional in their cellular locomotion and bone-resorption activity, which is associated with the disarrangement of podosomes and adhesion complex molecules including Pyk2. Such an experimental model exhibited an osteoporosis-resistance induced by RANKL (119). These data are in line with a previous study showing the CCR5-antagonist Maraviroc associated with a lower degree of bone loss in the hip and lumbar spine of HIV-infected individuals, as an example of a CCR5-antagonist treatment that might help to improve bone health among HIV-infected patients (120).

In conclusion, important progress has been made in our understanding of the effect of antiretroviral drugs on bone health in HIV-infected people. Such advances have enriched our ability to apply treatment to diminish aging-associated complications, such as osteoporosis and fractures.

## Concluding Remarks

During HIV infection and its progression to AIDS, bone loss occurs and HAART likely contributes -at least in part- to this comorbidity, involving both factors associated with disease reversal and direct skeletal effects. Although the clinical and imaging characterization of HIV bone pathology has been well-documented, the pathogenic mechanisms of bone loss have only been partially elucidated at present.

Irrespective of the mechanisms involved, diagnostic and therapeutic measures are necessary to delay the onset of bone disease in HIV patients to prevent a significant new threat to the health of the HIV/AIDS population.

## Author Contributions

MD and JQ conceived the idea and drafted the manuscript. All authors contributed to the article and approved the submitted version.

## Conflict of Interest

The authors declare that the research was conducted in the absence of any commercial or financial relationships that could be construed as a potential conflict of interest.
